# Temporal and spatial changes of macrobenthos community in the regions frequently occurring black water aggregation in Lake Taihu

**DOI:** 10.1038/s41598-018-24058-y

**Published:** 2018-04-09

**Authors:** Jianqin Chen, Dongfang Hu, Chenling Zhang, Zhengfeng Ding

**Affiliations:** Jiangsu Key Laboratory of Biofunctional Molecule, School of Life Sciences, Chemistry & Chemical Engineering, Jiangsu Second Normal University, 77 West Beijing Road, Nanjing, 210013 China

## Abstract

Seasonal survey was performed from August 2015 to May 2016 at 50 sampling sites in Lake Taihu to determine the spatial and temporal changes in macrobenthos community and their relationships with environmental variables. A total of 58 macrobenthos species were collected and identified, including 28 species of annelids, 17 species of molluscs, and 12 species of arthropods. Both the community composition and the dominant species changed temporally and spatially. Correspondingly, the macrobenthos biodiversity differed among regions and seasons. The macrobenthos density decreased with increased sediment depth, which is the first report about the vertical distribution of macrobenthos in Lake Taihu. The majority of benthic animals were located within the sediment depth of 0–5 cm and 5–10 cm, accounting for 39.25% and 24.87% of the total abundance respectively. Redundancy discriminate analysis revealed that the main environmental factors affecting the most contributing macrobenthos species were temperature in summer, transparency, dissolved oxygen and pH in autumn, and water depth and dissolved oxygen in winter. Particularly, salinity and conductivity showed high correlation with the macrobenthos community through the whole sampling period. The investigation reveals the inherent spatiotemporal variation of macrobenthos community, and provides references for the biological assessment of water quality in Lake Taihu.

## Introduction

Aquatic macrobenthos make up a large component of benthic community, and mediate the ecosystem processes such as the sediment decomposition^1,2^. Combined with their relatively sedentary lifestyles such as long life-cycles and poor mobility, macrobenthos respond to environmental changes via community-related variations including species composition, diversity, abundance, and biomass^3–6^. It is widely studied that the changes in community structure of macrobenthos correlated well with the variations in environmental factors^7–10^. Thus, macrobenthos commonly serve as useful bio-indicators for aquatic environment monitoring and assessment in river, marine and lake ecosystem^11–15^.

Lake Taihu, located in the Yangtze delta region, is the third largest freshwater lake in China. As a result of the increased nutrient input, Lake Taihu has undergone rapid eutrophication with frequent outbreak of cyanobacteria blooms^16^. Because Lake Taihu contributes a great deal to the aquatic products in China, and is also an important drinking water source for the surrounding densely cities, several investigations of water quality based on benthic invertebrate have been carried out to provide information for the understanding of lake environments^17–19^. In a recent study, based on the two-year investigation from February 2007 to November 2008, Cai *et al*.^20^ determined the dominant species, e.g. *Limnodrilus hoffmeisteri*, *Rhyacodrilus sinicus, Corbicula fluminea, Bellamya aeruginosa, Tanypus chinensis*, and *Gammarus* sp. in Lake Taihu, and the critical environmental factors of trophic level, sediment type and the distribution of aquatic vegetation. Xie *et al*.^21^ studied that there were a total of 42 macrobenthos species with *C. fluminea, L. hoffmeisteri and Tubificidae* sp. as the indicator species of the whole lake from the winter of 2010 to the autumn of 2012, and the main abiotic factors affecting the benthic community varied depending on the districts of the lake. Previous investigations on the macrobenthos in Lake Taihu demonstrated that there were differences in the inherent spatial and temporal distribution of macrobenthos community, which is further affected by the environmental disturbance^22,23^.

Black water aggregation, which is characterized by black color, offensive odor, and low dissolved oxygen (<1 mg L^−1^) in the water column, often forms after severe cyanobacterial blooms. It poses severe threats to the drinking water safety. With the aggravation of cyanobacteria bloom, black water aggregation has occurred frequently in many inland lakes and continental seas, such as Florida Keys in USA^24^, East China sea^25^ and Baltic in Europe^26^. The black water aggregation in Lake Taihu mostly occurs in areas with high levels of polluted sludge and accumulated cyanobacteria^27,28^. The occurrence of black water aggregation generally accompanied with the deterioration of water quality, which potentially alters the habitat of macrobenthos. In this study, we aimed to analyze the spatial and temporal variations in the community structure of the macrobenthos in black water aggregation districts of Lake Taihu, namely the West basin of Lake Taihu, Meiliang Bay, Zhushan Bay, and Gonghu Bay from August 2015 to May 2016. The main environmental factors affecting the macrobenthos community were also determined.

## Results

### Species composition and biodiversity of macrobenthos

There were total 55386 individuals collected during the sampling period, which were identified to 58 species and belonged to 4 phylum, 7 class, 15 families, 25 genera. Among these species, 28 were classified as annelids, making up 48.26% of all the individuals; 17 belonged to molluscs accounting for 29.31%, and 12 were arthropods (20.69%). The widespread species (occurrence frequency > 50%) includes *Branchiura sowerbyi*, *Tubifex tubife*, *Limnodrilus hoffmeisteri*, *Tanypus punctipennis*, and *Microchironomus tener*. The detailed information of species distribution can be found in Table 1.

**Table 1:** Macrozoobenthos distribution in black water aggregation areas in Lake Taihu

No	Species	DGH	WXG	DPG	TG	ZHG	LXH	XXG	XD	WYH
	**Nematomorpha**									
1	*Nematomorpha* sp.		+				+			
	**Annelida**									
	**Nereididae**									
2	*N. Aibiuma*		+	+		+	+		+	+
	**Nephtyidae**									
	***Nephtys***									
3	*N. polybranchia*			+	+	+	+	+	+	+
	**Capitellidae**									
	***Capitella***									
4	*Capitellidae* sp.		+	+		+	+	+	+	+
	**Sabellinae**									
	**Chone**									
5	*Chone* sp.		+	+	+	+	+	+	+	+
	**Naididae**									
	***Nais***									
6	*N. variabilis*	+	+	+	+	+	+	+	+	
	***Slavina***									
7	*S. appendiculata*						+			
	***Pristina***									
8	*Pristina* sp.		+	+		+				
	***Dero***									
9	*D. digitata*	+	+	+	+	+	+	+	+	
10	*D. obtusa*	+	+	+		+	+			
11	*D. austrina*	+	+	+		+	+			
	**Tubificidae**									
	***Branchiura***									
12	*B. sowerbyi*	+	+	+	+	+	+	+	+	+
	***Tubifex***									
13	*T. tubifex*	+	+	+	+	+	+	+	+	+
14	*Rhyacodrilus sinicus*	+	+	+	+	+	+	+	+	+
15	*T. sinicus*	+	+	+	+		+		+	
	***Aulodrilus***									
16	*A. prothecatus*	+	+	+						
	***Limnodrilus***									
17	*L. hoffmeisteri*	+	+	+	+	+	+	+	+	+
18	*L. claparedianus*	+	+							
19	*L. grandisetosus*	+	+	+	+	+	+	+	+	+
	**Glossiphoniidae**									
	***Glossiphonia***									
20	*G. complanata*	+			+					
21	*G. lata*		+		+					
22	*G. weberi*				+					
23	*G. heteroclita*		+		+					
	***Batracobdella***									
24	*B. kasmiana*			+	+					
	***Helobdella***									
25	*H. stagnalis*		+		+	+				
26	*H. nuda*		+	+	+	+		+	+	
	***Placobdella***									
27	*P. okadai*			+						
	**Ozobranchidae**									
	***Ozobranchus***									
28	*Ozobranchus* sp.			+						+
	**Erpobdellidae**									
	***Erpobdella***									
29	*E*. o*ctoculata*		+	+						
	**Mollusca**									
	**Viviparidae**									
	***Bellamya***									
30	*B. aeruginosa*	+	+	+	+	+	+	+	+	+
31	*B. purificata*				+		+			
32	*B. quadrata*									+
	**Hydrobiidae**									
	***Stenothyra***									
33	*S. glabra*	+	+	+	+	+	+		+	+
	***Alocinma***									
34	*A. longicornis*		+	+	+					
	***Parafossarulus***									
35	*P. striatulus*				+					+
	***Bithynia***									
36	*B. fuchsiana*				+					+
	**Melaniidae**									
	***Semisulcospira***									
37	*S. cancellata*		+	+						
	**Lymnaeidae**									
	***Radix***									
38	*R. clessini*				+					
	**Mytilidae**									
	***Limnoperna***									
39	*L. lacustre*	+			+					
	**Solecurtidae**									
	***Novaculina***									
40	*N. chinensis*		+							
	**Unionidae**									
	***Anodonta***									
41	*A. angula*	+		+	+	+	+		+	
42	*A. arcaeformis*		+							
	***Lanceolaria***									
43	*L. gladiolus*		+	+	+					
	***Arconaia***									
44	*A. lanceolaia*					+			+	+
	**Corbiculidae**									
	***Corbicula***									
45	*C. fluminea*	+	+	+	+	+	+	+	+	+
	***Sphaerium***									
46	*S. lacustre*	+	+	+	+	+				
	**Arthropoda**									
	**Caenidae**									
47	*Caenidae*. sp	+								
	**Chironomidae**									
	**Tanypodinae**									
	***Tanypus***									
48	*T. punctipennis*	+	+	+	+	+	+	+	+	+
	**Orthocladiinae**									
	***Propsilocerus***									
49	*P. akamusi*	+	+	+	+	+	+	+	+	+
	**Chironominae**									
	***Chironomus***									
50	*C. plumosus*	+	+	+	+	+	+	+	+	+
	***Microchironomus***									
51	*M. tener*	+	+	+	+	+	+	+	+	+
	***Cryptochironomus***									
52	*Cryptochironomus* sp.	+	+	+	+	+	+	+	+	+
	**Muscidae**									
53	*Muscidae* sp.		+							+
	**Libellulidae**									
	***Libellula***									
54	*Libellulidae* sp.		+		+					
	**Oedicerotidae**									
	***Monoculodes***									
55	*M. limnophilus*	+	+	+	+	+	+	+	+	+
	**Corophiidae**									
	***Grandidierella***									
56	*G. aihuensis*	+	+	+	+	+	+	+	+	+
	**Ingolfiellidae**									
	***Paranthura***									
57	*P. japonica*	+	+	+		+	+			
	**Palaemonidae**									
	**Macrobrachium**									
58	*M. nipponense*		+	+	+					

Note: DGH-Dagang River; WXG-Wuxi harbor; DPG-Dapu harbor; TG-Taige canal; ZHG-Zhihu harbor; LXH-Liangxi River; XXG-Xiaoxi harbor; XD-Wuxi sewage treatment plant; WYH-Wangyu River. “+”Denotes that the species was found in this sampling site.

**Figure 1:**
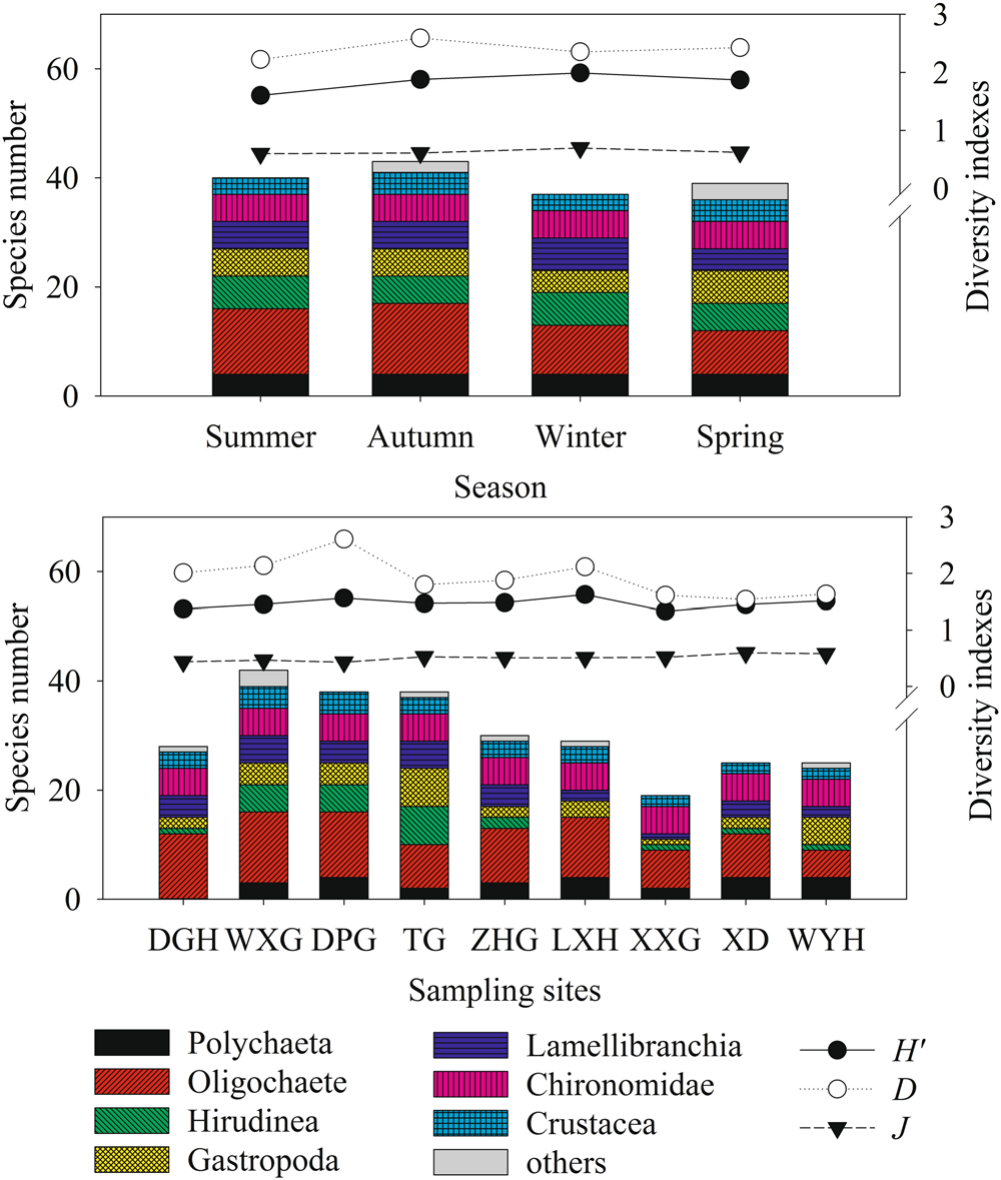
Seasonal and horizontal variations in species number and diversity indexesof macrozoobenthos in black water aggregation regions in Lake Taihu. Note: DGH-Dagang River; WXG-Wuxi harbor; DPG-Dapu harbor; TG-Taige canal; ZHG-Zhihu harbor; LXH-Liangxi River; XXG-Xiaoxi harbor; XD-Wuxi sewage treatment plant; WYH-Wangyu River. The seasonal and horizontal difference in species number was detected by Kruskal-Wallis H test. The seasonal and horizontal difference in diversity indexes was analyzed by ANOVA.

The species composition of macrobenthos community varied temporally and spatially (Fig. 1). There was the highest species number of 43 in autumn, with the lowest 37 in winter. Specifically, there were significantly seasonal changes in the species number belonging to Oligochaete (*χ*_3_^2^ = 20.479, *P* < 0.05), Chironomidae larvae (*χ*_3_^2^ = 41.052, *P* < 0.05), and Crustacea (*χ*_3_^2^ = 28.049, *P* < 0.05). Referring to the spatial difference, the number of macrobenthos species in West basin of lake and Zhushan Bay was the largest, with the lowest in Gonghu Bay (Fig. 1). There were as many as 42 species in sampling sections near the Wuxi harbor, and only 19 species in sampling sections near the Xiaoxi harbor. The species with significant changes among different sampling sections belonged to *Polychaete*, *Oligochaete*, *Hirudinea*, *gastropoda*, *Lamellibranchia*, *Chironomidae*, and *Crustacea*.

The biodiversity indexes of the macrobenthos kept in a range of 1.39–2.39 for Shannon-Weiner diversity index (*H’*), 1.59–3.52 for Margalef richness index (*D*) and 0.46–0.84 for Pielou evenness index (*J’*). The Xiaoxi harbor in summer has the lowest values of *H’* and *D*, the highest values of which appeared in Wangyu River in winter and Dapu harbor in summer. The values of *J’* reached its peak in Wangyu River in winter, and has the lowest value in Dagang River in summer. Seasons significantly affected the *H’* (*F*_3*,194*_ = 4.093, *P* < 0.05) and the *J’* (*F*_3*,194*_ = 2.874, *P* < 0.05), but not the *D* (*F*_3*,194*_ = 2.076, *P* > 0.05). In particular, the *H’* of macrobenthos were significantly different between summer and winter, and the *J’* differed between autumn and winter (Fig. 1).

Referring to the spatial variations, no significant difference was found in *H’* (*F*_*8,189*_ = 0.655, *P* > 0.05) among the sampling sections, but the *D* (F_8,189_ = 9.833, *P* < 0.05) and *J’* (*F*_*8,189*_ = 3.690, *P* < 0.05) significantly changed with sampling sections. Tukey’s multiple comparison analysis indicated significant differences in the *D* between DPG and WXG, TG, ZHG, XXG, XD, WYH, between WXG and XXG, XD, WYH, and between LXH and XD. The *J’* was significantly different between DGH and XD, and between DPG and XD and WYH (Fig. 1).

### Dominant species

The dominant macrobenthos species in black water aggregation regions in Lake Taihu varied depending on season and location. There were total 8 dominant species, among which, *T. tubife*, *L. hoffmeistteri*, *Bellamya aeruginos*a, *Corbicula fluminea*, and *T. punctipennis* were detected during the whole sampling period, and *L. grandisetosus* and *Propsilocerus akamusi* were dominated only in winter, with *B. purificata* only in summer (Table 2). In the spatial scale, the dominant species varied among sampling sections (Table 3). In particular, *L. hoffmeistteri* is always the dominant species in all the nine sampling sections, and *T. punctipennis* dominated only in the sections adjacent to Liangxi River and Xiaoxi harbor. *Propsilocerus akamusi* was only observed in the section adjacent to Liangxi River. The detailed dominance and horizontal or seasonal variations of the dominated species in different seasons and sampling locations can be found in Tables 2 and 3.

**Table 2:** The dominance (*IRI*) and seasonal variations of the dominant macrozoobenthos in black water aggregation areas in Lake Taihu.

Dominated species	Summer				Autumn				Winter				Spring			
	West basin	Zhushan Bay	Meiliang Bay	Gonghu Bay	West basin	Zhushan Bay	Meiliang Bay	Gonghu Bay	West basin	Zhushan Bay	Meiliang Bay	Gonghu Bay	West basin	Zhushan Bay	Meiliang Bay	Gonghu Bay
*Tubifex tubife*	1181.71	—	—	1439.55	1490.25	3376.72	1974.04	3988.61	1643.44	—	1585.15	2055.33	1547.82	—	—	—
*Limnodrilus hoffmeistteri*	4086.71	7594.06	4854.93	—	5732.27	4324.84	6253.40	3180.14	6037.29	5280.58	3559.95	1575.34	5914.20	3200.50	4764.06	3605.86
*Limnodrilus grandisetosus*	—	—	—	—	—	—	—	—	—	1158.80	—	—	—	—	—	—
*Bellamya aeruginosa*	—	3035.95	—	—	—	5072.93	1349.81	—	—	1250.11	—	—	—	1826.02	1613.17	—
*Bellamya purificata*	—	1055.22	—	—	—	—	—	—	—	—	—	—	—	—	—	—
*Corbicula fluminea*	4065.52	—	1688.58	—	4629.35	—	—	—	6922.17	2123.34	1185.43	—	6992.22	1247.60	—	—
*Tanypus punctipennis*	—	—	—	1192.71	—	—	1805.22	—	—	—	1272.93	1046.88	—	2759.40	2412.97	2212.62
*Propsilocerus akamusi*	—	—	—	—	—	—	—	—	—	—	2677.58	3262.89	—	—	—	—

**Table 3:** The dominance (*IRI*) and horizontal distribution of the dominant macrozoobenthos in black water aggregation areas in Lake Taihu.

Dominated species	Dagang river	Wuxi harbor	Dapu harbor	Taige canal	Zhihu harbor	Liangxi river	Xiaoxi harbor	Wuxi plant	Wangyu river
*Tubifex tubife*	2468.92	1430.34	—	—	1528.51	—	—	2562.78	3093.78
*Limnodrilus hoffmeistteri*	4714.45	4688.39	6263.20	5690.36	5491.69	3975.81	2848.97	2062.46	1377.49
*Bellamya aeruginosa*	—	—	—	2624.01	—	1796.13	1819.77	—	—
*Corbicula fluminea*	5767.80	—	7379.65	1060.48	1865.08	—	—	—	—
*Tanypus punctipennis*	—	—	—	—	—	2746.47	2732.41	—	—
*Propsilocerus akamusi*	—	—	—	—	—	1381.53	—	—	—

### Temporal and spatial variation of density and biomass of macrobenthos

**Figure 2:**
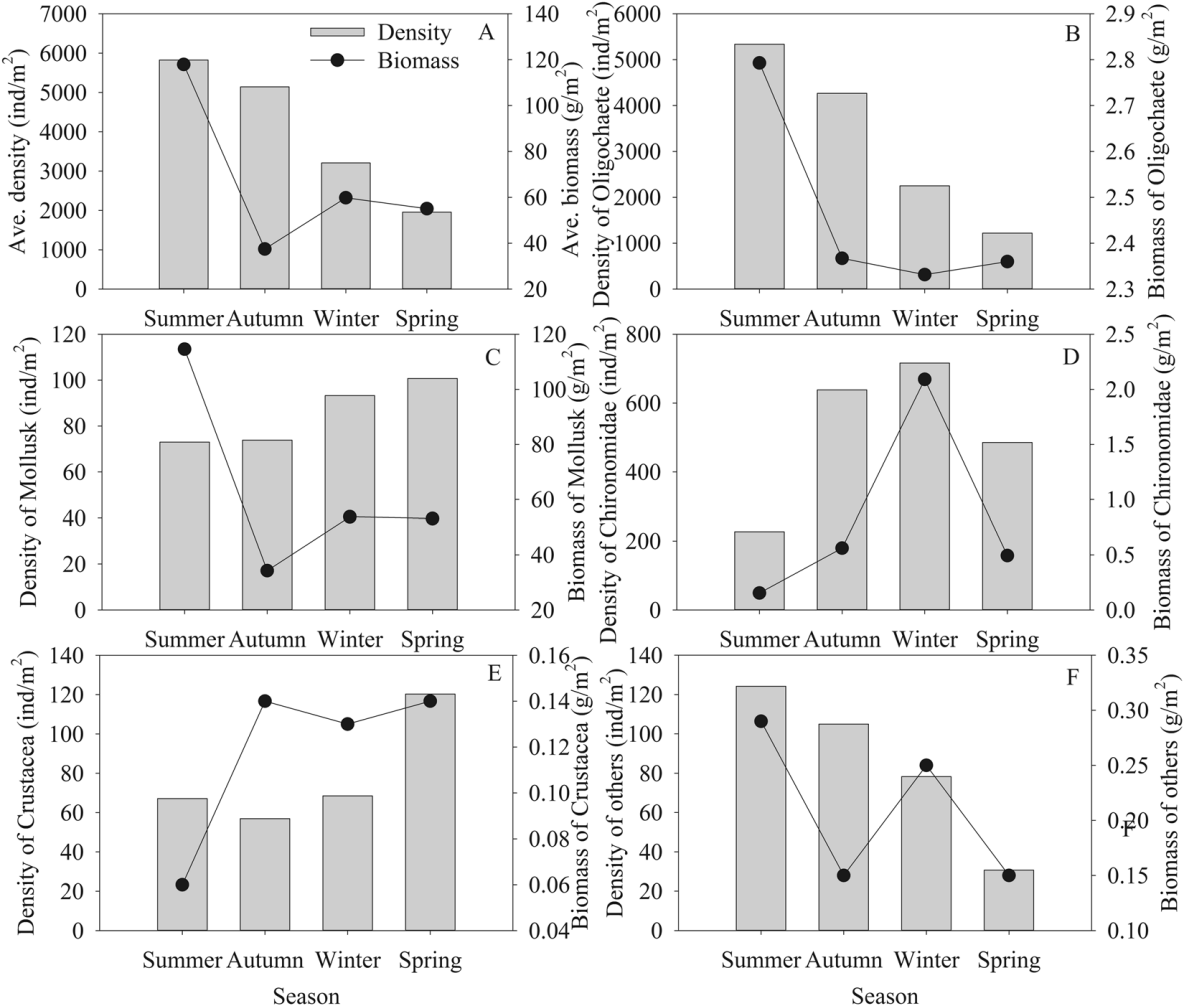
Seasonal variation in average density and biomass (**A**) of macrobenthos including Oligochaeta (**B**), Mollusk (**C**), Chironomidae (**D**), Crustacea (**E**) and others **(F**) in the black water aggregation regions in Lake Taihu. The seasonal difference in species’ density and biomass was determined by Kruskal-Wallis H test.

The macrobenthos density averaged 4015.17 ± 447.39 ind/m^2^ across the four seasons, with the highest mean density of 5826.33 ± 1373.26 ind/m^2^ in summer and the lowest 1958.83 ± 256.32 ind/m^2^ in spring. The Kruskal-Wallis H test revealed no significant difference among the macrobenthos density in different seasons (*χ*_3_^2^ = 5.000, *P* > 0.05). Oligochaete (3247.56 ± 415.08 ind/m^2^) and Chironomidae (519.84 ± 71.01 ind/m^2^) had the highest densities. Correspondingly, the macrobenthos biomass was comparable in temporal scale (*χ*_3_^2^ = 1.035, *P* > 0.05), with the average biomass of 66.74 ± 12.23 g/m^2^. Specifically, the mollusk (63.42 ± 12.08 g/m^2^), Oligochaete (2.17 ± 0.30 g/m^2^), Chironomidae (0.83 ± 0.19 g/m^2^), Crustacea (0.13 ± 0.03 g/m^2^) and Polychaete (0.17 ± 0.04 g/m^2^) contributed to the majority of the biomass. The Kruskal-Wallis H test revealed no difference among the seasonal biomasses in Oligochaete (*χ*_3_^2^ = 3.110, *P* > 0.05) and Mollusk (*χ*_*3*_^2^ = 4.707, *P* > 0.05), but significant differences in Chironomidae (*χ*_*3*_^2^ = 21.451, *P* < 0.05) and Crustacea (*χ*_*3*_^2^ = 22.333, *P* < 0.05) (Fig. 2).

**Figure 3:**
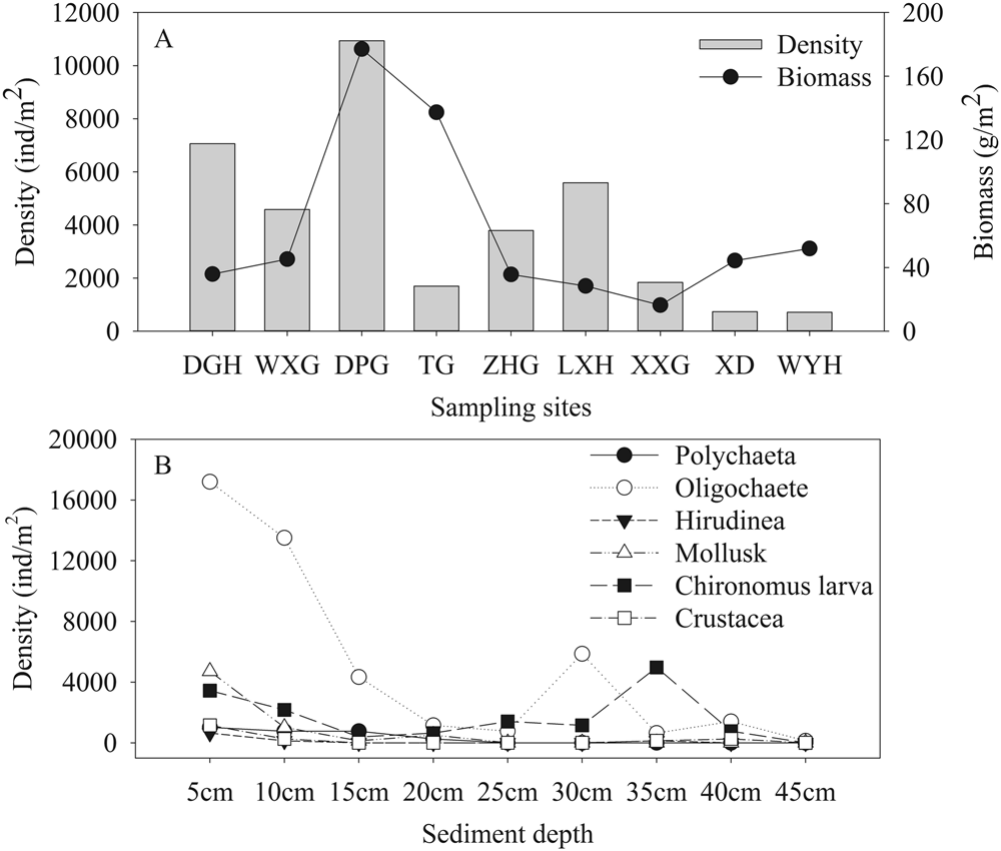
Average density and biomass in black water aggregation regions (**A**) and vertical density distribution in Moon bay (**B**) of macrozoobenthos in Lake Taihu. Note: DGH-Dagang River; WXG-Wuxi harbor; DPG-Dapu harbor; TG-Taige canal; ZHG-Zhihu harbor; LXH-Liangxi River; XXG-Xiaoxi harbor; XD-Wuxi sewage treatment plant; WYH-Wangyu River. The average density and biomass in black water aggregation regions was calculated as the mean values of those in the sampling four seasons. ANOVA and Kruskal-Wallis H test were used to analysis the differences in average density and biomass respectively.

The macrobenthos density (*F*_8,189_ = 9.650, *P* < 0.05) and biomass (*χ*_8_^2^ = 51.355, *P* < 0.05) also varied with spatial locations (Fig. 3A). The DPG section had the highest macrobenthos density (10938.78 ± 2470.86 ind/m^2^), which was followed by DGH section (7060.63 ± 1235.91 ind/m^2^). The WYH section had the smallest value of macrobenthos density (713.44 ± 124.15 ind/m^2^). Corresponding to the highest density, DPG section had the highest biomass of 177.07 ± 63.98 g/m^2^ in contrast to the lowest biomass of 16.38 ± 8.34 g/m^2^ in XXG section.

There were 22 macrobenthos collected during the vertical sampling in Moon bay in spring 2015, of which 19 species were located within 0–5 cm of the sediment (Fig. 3B). The macrobenthos density generally decreased with increased sediment depth. The benthic animals located within the sediment depth of 0–5 cm and 5–10 cm accounted for 39.25% and 24.87% of the total abundance respectively. The vertical distribution of macrobenthos species were highly affected by the sediment depth. The Polychaeta and Mollusk distributed within 0–20 cm of the sediment. Hirudinea distributed within 0–10 cm of the sediment in all sampling sites except for the YA-7 station. Crustacea were also found within 0–10 cm of the sediment except for the YA-5 sampling point. *Chironomus* larvae and Oligochaete widely distributed within 0–40 cm and 0–45 cm of the sediment.

### Physiochemical parameters and their relationship with macrobenthos community

The physicochemical parameters while sampling were presented in Table 4. The water depth was relatively shallow in winter and spring. The water pH, L and MD at different sampling sites were comparable in the temporal scale. The WT in spring and summer (23.81–28.69 °C) was significantly higher than that in autumn and winter (10.16–13.72 °C). The Eh reached peak (247.74 mv) in spring with the lowest value of 70.82 mv in autumn. The average DO decreased by ~30% in summer compared with the constant DO in other seasons. The SD in autumn and winter was significantly higher than that in spring and summer. Both the COND and SAL reached peak values (668.88 *μ*S·cm^−1^ for COND and 0.33 mg L^−1^ for SAL) in winter with the low values in autumn. The TDS fluctuated from 294.24 mg L^−1^ in autumn to 456.38 mg L^−1^ in winter.

**Table 4:** Environmental factors of black water aggregation areas in Lake Taihu.

Season		T/°C	WD/m	pH	WT/°C	Eh/mv	DO/mg·L^−1^	SD/m	COND *μ*S·cm^−1^	SAL/mg·L^−1^	TDS/mg·L^−1^	L/m	MD/m
Summer	maximum	34.20	3.70	8.74	30.50	246.20	13.83	0.40	770.00	0.38	527.00	6110.00	0.85
	minimum	23.60	1.00	6.92	25.60	29.60	0.98	0.08	169.00	0.08	112.00	0.00	0.00
	average	28.95	2.31	7.61	28.69	113.84	6.96	0.27	573.38	0.28	389.81	1547.38	0.29
Autumn	maximum	10.00	4.50	8.78	11.50	97.00	11.74	0.85	563.00	0.30	421.00	6110.00	0.85
	minimum	10.00	0.60	7.52	9.00	40.00	2.67	0.18	173.90	0.08	115.00	0.00	0.00
	average	10.00	2.29	8.20	10.16	70.82	9.90	0.48	431.40	0.21	294.24	1571.38	0.29
Winter	maximum	15	4.20	9.50	15.80	153.00	11.77	0.80	901.00	0.44	621.00	6110.00	0.85
	minimum	10	0.40	5.00	12.80	55.00	8.07	0.20	516.00	0.25	350.00	0.00	0.00
	average	11.7	1.94	8.52	13.72	95.12	9.72	0.42	668.88	0.33	456.38	1571.38	0.29
Spring	maximum	30.00	4.20	9.32	26.96	375.80	27.32	0.40	732.00	0.37	489.00	6110.00	0.85
	minimum	24.00	0.40	6.97	21.94	39.60	1.60	0.00	54.00	0.12	167.00	0.00	0.00
	average	27.52	1.94	8.61	23.81	247.74	10.69	0.19	456.90	0.23	311.64	1571.38	0.29

Note: T-climate temperate; WD-water depth; WT-water temperature; Eh-oxidation-reduction potential; DO-dissolved oxygen; SD-secchi depth; COND-conductivity; SAL-salinity; TDS-total dissolved solid; L-the distance from the shore; MD-sediment thickness.

According to Pearson’s correlation test, different relationships between the macrobenthos species number, density, biomass and environmental factors in the four seasons were observed (Table 5): (1) the animal species number was positively correlated with the L in summer, with T and WD in winter, and with Eh in spring, but negatively correlated with SD in autumn; (2) the animals density was negatively related with the WT, COND, SAL, and TDS in summer, with SAL in autumn, and positively correlated with T and WD in winter. In spring, the macrobenthos density was negatively related with COND and SAL, but positively related with L; (3) No closely relationship between macrobenthos biomass and environmental factors was found in summer and spring. The biomass was negatively correlated with Eh, DO, pH and SD in autumn, but positively correlated with COND, SAL and TDS in winter.

**Table 5:** Correlation analysis of environmental factors with macrozoobenthos numbers, density and biomass in black water aggregation regions in Lake Taihu.

Season		T	WD	pH	WT	Eh	DO	SD	COND	SAL	TDS	L	MD
Summer	species	0.010	0.075	−0.188	−0.114	−0.006	−0.109	0.023	0.006	−0.046	0.018	0.348*	0.115
	density	−0.012	−0.088	−0.181	−0.305*	−0.038	−0.093	−0.136	−0.298*	−0.306*	−0.291*	0.264	0.020
	biomass	−0.162	−0.133	−0.245	−0.252	0.043	−0.147	0.071	−0.051	−0.057	−0.044	0.104	−0.035
Autumn	species	0.025	−0.019	−179	−0.039	−0.223	0.038	−0.440**	−0.107	−0.199	−0.111	0.174	0.238
	density	0.137	0.168	−0.116	−0.260	0.247	0.247	−0.270	−0.205	−0.291*	−0.224	0.232	0.196
	biomass	0.152	−0.127	−0.595**	0.189	−0.346*	−0.297*	−0.364**	0.197	0.204	0.255	0.101	0.164
Winter	species	0.369**	0.316*	0.071	0.013	0.057	−0.076	−0.147	−0.016	0.043	−0.009	0.125	0.137
	density	0.300*	0.357*	0.141	0.110	0.110	−0.061	−0.208	−0.214	−0.189	−0.216	0.019	0.160
	biomass	0.246	0.144	−0.073	0.207	−0.190	−0.392**	−0.215	0.280*	0.297*	0.287*	0.222	−0.069
Spring	species	0.155	−0.051	0.199	0.146	0.303*	0.238	−0.200	−0.201	−0.255	−0.202	0.242	0.017
	density	0.226	0.141	0.210	0.240	0.217	0.220	−0.154	−0.284*	−0.305*	−0.268	0.334*	0.133
	biomass	0.011	0.278	0.006	0.218	0.147	0.093	−0.027	−0.232	−0.100	−0.125	0.171	−0.179

### Correlation of environmental factors with the dominant species

**Figure 4:**
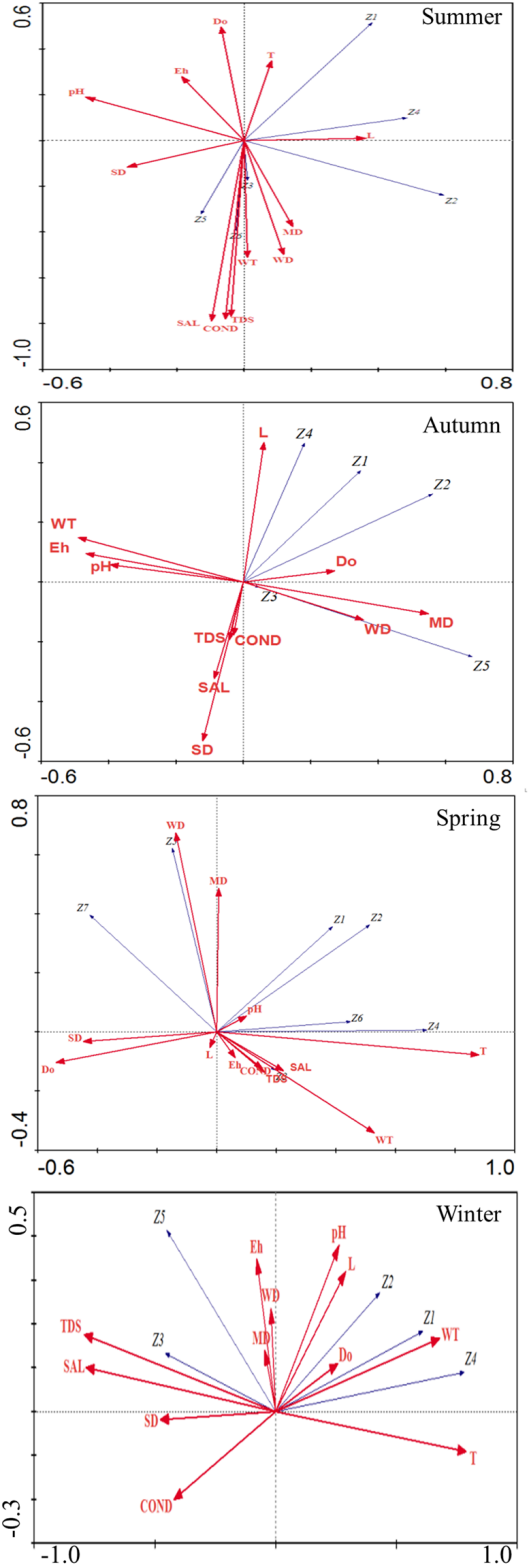
The Redundancy analysis of macrobenthos and environmental factors in four seasons. Note: Z1-*T. tubifex*; Z2-*L. hoffmeisteri*, Z3-*B. aeruginosa*, Z4-*C. fluminea*, Z5-*T. punctipennis*.

Based on the length <4 of the DCA axis, RDA was used to analyze the relations between dominant macrobenthos species and environmental factors (Fig. 4). In summer, the densities of dominated *B. aeruginosa*, *T. punctipennis*, *B. purificata* were positively related with SAL, COND, TDS, WT, WD, and MD, but was negatively related with Eh, DO, and T. The dominated *C. fluminea* and *T. tubifex* were positively related with L, but negatively related with SD. The dominated *L. hoffmeisteri* was positively related with L, MD, and WD, but negatively related with pH, Eh, and SD. In autumn, the dominated *T. tubifex* and *C. fluminea* were positively related with L and DO, and negatively related with COND, TDS, SAL, and SD. The dominated *L. hoffmeisteri*, *B. aeruginosa* and *T. punctipennis* were positively related with DO, MD and WD, and negatively related with WT, Eh, and pH.

In winter, the dominated *B. aeruginosa*, *C. fluminea* and *L. grandisetosus* were positively related with T, WT, SAL, TDS, COND, Eh and pH, and negatively related with SD and DO. The dominated *T. tubifex* and *L. hoffmeisteri* were positively related with MD and pH, and negatively related with SD, DO and L. The dominated *T. punctipennis* and *P. akamusi* were positively related with WD and MD, and negatively related with SAL, WT, TDS, COND and Eh. In spring, the dominated *T. tubifex*, *L. hoffmeisteri* and *C. fluminea* were positively related to WT, T, L, pH and DO, and negatively related to TDS, SAL, SD and COND. The dominated *B. aeruginosa* and *T. punctipennis* were positively related to TDS, SAL, SD, MD, WD and Eh, and negatively related to T.

## Discussion

The present investigation showed that the number of macrobenthos species, density and biomass changed among seasons, during which, the number of species reached the peak in autumn and the density and biomass were highest in summer. The macrobenthos in black water aggregation areas in Lake Taihu were dominated by the Oligochaeta and Chironomus larva, consisting of 93.83% of the total macrobenthos density. This is comparable to the finding by Cai *et al*.^20^ based on the quarterly investigation on macrozoobenthos between February 2007 and November 2008. The dominance by the resistant Oligochaeta and Chironomidae generally suggests the water deterioration^29–31^, which is in accordance with the declining water quality when black water aggregation occurs. While large amounts of cyanobacteria accumulated and died under certain conditions like high temperature, slow wind, and weak reoxygenation capacity, thioethers substances such as volatile sulfide were released after the decomposition of dead cyanobacteria. These substances could chemically combine other materials like the heavy metals from sediment, favoring the formation of black water aggregation and water deterioration^27,32,33^. In spite of high density of macrobenthos in autumn, the animals’ biomass was relatively low because of the small sizes of Oligochaeta and Chironomus larva. The more frequency of mollusks with high biomass resulted in the relatively high macrobenthos biomasses in total in other seasons (Figs 2 and 3).

The macrobenthos community also varied spatially. Among the nine sampling sections, Wuxi harbor had the maximum 42 macrobento species, contrasting to Xiaoxi harbor with the minimum 19 species. In particular, the sampling sections of Taige canal, Wuxi sewage treatment plant and Wangyu River had relatively higher biomasses although the macrobenthos density was low. This is attributed to the high abundance of mollusks in these sections (Fig. 1). Qiu *et al*.^34^ studied that the gastropod like the *B. aeruginosa* shifted its diets from the planktic to benthic materials under toxic cyanobacterial bloom. Thus, sandy silt and aquatic plants benefit the growth and reproduction of mollusk^35,36^, which was further evidenced by the silty sand substrate and several macrophyte distributed of Taige canal section dominated by *B. aeruginosa*. The sediment near to Wuxi sewage treatment plant and Wangyu River was covered by plenty of aquatic plants with rigid substrate because of the silt clearing. The missing silt results in the decreases of the density and biomass of Oligochaete including the Tubifex and Limnodrilus, whereas aquatic plants favor the growth of mollusk. The spatial differences in macrobenthos in sampling sections led to the diversity of macrobenthos in different regions of Lake Taihu. Based on our result, West basin of the lake had the highest macrobenthos density. Given the second high density of macrobenthos, the biomass in Meiliang Bay was the lowest with the highest abundance of Chironomidae larvae. This is in accordance with the investigation by Qin *et al*.^18^ and Cai *et al*.^20^. Because of the deep sediment (averaged > 1.5 m), the organic substances were high in Meiliang Bay. In addition, the cyanobacteria, together with other phytoplankton such as the diatom, tend to accumulate in this region with the decreased dissolution oxygen due to the southeast monsoon in summer. These environments benefit the growth of Chironomidae larvae. The dominance by *B. aeruginosa* in Gonghu Bay in 2010^20^ was not observed in present investigation, which was replaced by *T. tubife* and *L. hoffmeisteri*. The macrobenthos density was generally decreased from the offshore towards the centers of the Lake Taihu, which was comparable to the founding by Xu *et al*.^37^.

There were a few studies reporting the vertical distributions of macrobenthos in freshwater lakes except for these in intertidal zones or rivers with different altitudinal gradients^38–40^. Our study indicated that the macrobenthos in Lake Taihu distributed as deep as 45 cm in the sediment, which was significantly deeper than the 25 cm in Lake Donghu in China. Due to the high demands for dissolved oxygen and their filter-feeding behavior, Polychaeta and Mollusk dominated the upper sediment (0–20 cm). Chironomidae larvae and Oligochaete vertically distributed as deep as 45 cm in sediment, which may be highly related with their low dependence on oxygen and diving behavior to escape from the surface predation. The majority distribution of macrobenthos (88.28%) in upper sediments (0–30 cm) indicated that the oxygen and food resources may be the main environmental factors affecting the vertical distribution of macrobenthos in our study. Some other factors, such as organic matter and grain size, are also studied to regulate the vertical heterogeneous distribution of macrobenthic community^41,42^.

The main environmental factors affecting the macrobenthos community changed seasonally, which were water temperature in summer, transparency, dissolved oxygen, and pH in autumn, and water depth and dissolved oxygen in winter (Table 5). The varied sensitivities of benthic animals to physiochemical variables contributed to the temporal changes of main environmental factors. For example, the somatic growth and survival of *C. fluminea* were primarily determined by water temperature^43^. In particular, salinity and conductivity showed highly correlation with the benthic animals through the four seasons. The structuring factors of the macrobenthos community varied based on the numerous studies, for example, the depth in estuary and the sediment quality in deep sea^44–46^. Gao *et al*.^47^ studied that water conductivity, along with total nitrogen, were the main environmental factors affecting the distribution of macrobenthos in Lake Taihu from August 2009 to May 2010. The conductivity tended to influence the Oligochaeta and Mollusks mostly^48^, which was in accordance with the present study. Change in water quantity is one major reason affecting the salinity fluctuations. Because of the low rainfall and runoff in winter^49^, Lake Taihu had the highest salinity in winter. Rainfall generally increased from Spring. Nonetheless, evaporation also increased corresponding to the increased temperature, which reached peak in summer^50^. This may contributed to the relatively higher salinity in summer than those in spring and autumn. Different freshwater species showed unequal resistance to salinity fluctuation^51^. Besides, the adult species have generally greater osmoregulatory capability than their larvaes do^52^. The varied salinity-tolerances facilitated the seasonal changes of dominant macrobenthos species corresponding to the salinity fluctuation. Besides, the macrobenthos distribution was highly affected by many other abiotic variables like the nutrient-related factors. The diversity of macrobenthos was generally negatively related with the total nitrogen and total phosphorus. Moreover, Lake Taihu is kind of lakes for fish farming. The predation risk from fishes also influence the macrobenthos community^53^. Thus, more parameters need to be included in future investigations to better understand the biological-environmental relationships.

The habitat heterogeneity is traditionally considered to affect the biological structure^54,55^. Researchers have concluded that habitat complexity is the key variable determining the diversity of zoobenthos community^54,56^. Compared with the present macrobenthos structure in Lake Taihu including 58 species dominated by annelida, mollusks and arthropods, different benthic community structures also located in Yangtze River basin were observed. The Qingjiang River, which is located in the middle reaches of the Yangtze River, has as many as 82 zoobenthos species with the Shannon-Wiener index of 4.36^57^. In the lower reaches of Yangtze River, the water quality of Liangtang River and Dianshan Lake fell in the moderate to seriously polluted status with as little as 10 benthic species and the Shannon-Wiener index of 0.32^58^. In these waters, the benthic community was dominated by the pollution-tolerant species like the *L. hoffmeistteri* and *B. aeruginosa*. Although the water quality was not detected in our investigation, the similar dominated species suggest that Lake Taihu was still with the deterioration of water quality.

## Methods

### Sampling sites

**Figure 5:**
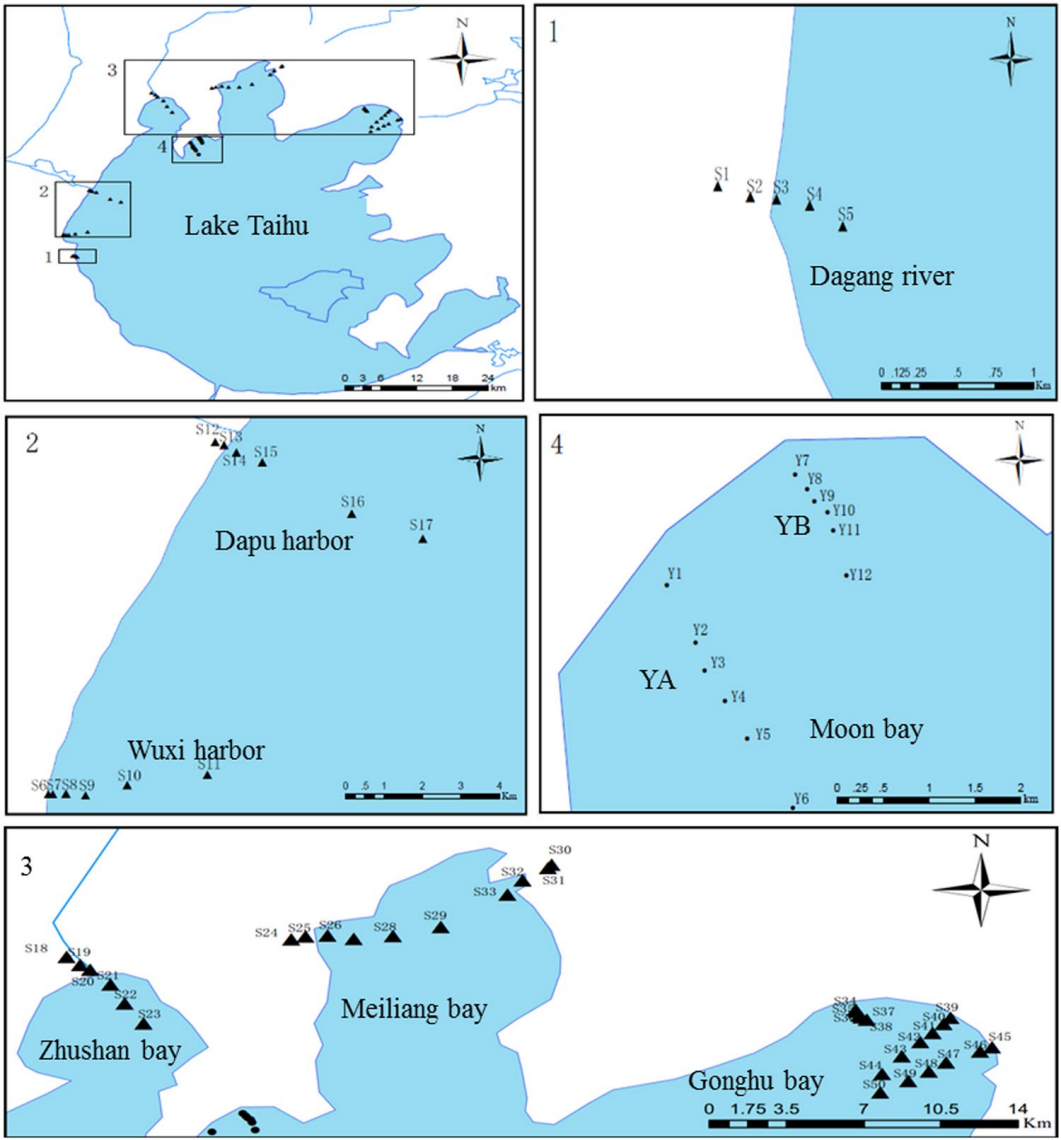
Overview and detailed map of the Lake Taihu with sampling sites. The blue lines represent the reviver into Lake Taihu. Triangles and circles represent the horizontal and vertical sites sampling respectively.

Four black water aggregation regions, namely the West basin of lake, Meiliang Bay, Zhushan Bay, and Gonghu Bay were investigated for the horizontal sampling. A total of 50 sampling sites were established from the inlet of rivers or domestic sewage treatment plant towards the lake center, including (1) 5 sites (S1-S5) near the inlet of Dagang River, 6 sites (S6-S11) near inlet of Wuxi harbor, and 6 sites (S12-S17) near the Dapu harbor in the West basin of lake; (2) 6 sites (S18-S23) near the Taige canal in Zhushan Bay; (3) 6 sites (S24-S29) near the Zhihu harbor, and 4 sites (S30-S33) near the Liangxi River in Meiliang Bay; (4) 5 sites (S34-S38) near the Xiaoxi harbor, 6 sites (S39-S44) near the Wangyu River, and 6 sites (S45-S50) near the Wuxi sewage treatment plant in Gonghu Bay. For the vertical sampling, two sampling sections including a total of 12 sampling sites (Y1-Y6 and Y7-Y12) were established in Moon Bay. The specific locations of these sampling sites are shown in Fig. 5.

### Sample collection

The horizontal sites sampling were carried out in August (autumn), November (winter) 2015 and February (spring), May (summer) 2016. Samples at each horizontal site were collected in three replicates together via a 1/16 m^2^ Peterson sediment sampler. The vertical sites sampling were carried out in May 2015 using a tube sampler with 10 cm in internal diameter. The tube sampler was manually sunk into the sediment to obtain sample as deep as 45 cm from the surface sediment. The cylindrically shaped samples at each site were immediately sectioned with stainless plate at an interval of 5 cm, and then stored in sampling bags respectively. All the horizontal and vertical samples were sieved through 60 unit mesh sieve at the site and preserved by adding 75% ethanol in 500-mL plastic bottles. The biological samples were then brought back to the laboratory and further fixed by adding 4% formaldehyde solutions^59^, which were sorted, enumerated and identified. The identification of individual specimens was preformed referring to the literatures^60–63^.

The physico-chemical parameters while sampling sediments were measured, including dissolved oxygen (DO) and water depth (WD) detected by portable dissolved oxygen meter (USA YSI-550A), and water temperature (WT), salinity (SAL), total dissolved solid (TDS), conductivity (COND), oxidation-reduction potential (ORP or Eh) and pH measured by water quality analyzer (China Y2001). The water transparency (indicated by secchi depth (SD)) was measured using a Secchi disk. The distance from the shore (L) was determined using Hand-held Laser Distance Meters.

### Data processing

The dominant species was determined based on the relative importance index (*IRI*), which was calculated as *IRI* = *(W* + *N)* × *F*, where *W* and *N* represents the biomass percent and abundance percent of one species, and *F* represents the frequency of occurrence percentage^64^. The species with *IRI* > 1000 was defined as the dominant species.

The diversity of macrobenthos was evaluated by Shannon-Wiener diversity index (*H*′), Margalef richness index (*D*), and Pielou evenness index (*J*′) as *H*′ = −Σ(*Pi*)(log_2_*Pi*); *D* = (*S* − 1) ln *N*; *J*′ = *H*′*/log*_2_*S*. *Pi* represents the abundance percent of the species *i*; *S* represents the species number, and *N* represents the abundance of total species.

The analysis of variance (ANOVA) or Kruskal-Wallis H was used to compare the difference in terms of species number, density, biomass and diversity among different sampling sections or among seasons. Kolmogorov-Smirnov was used to validate the normality. ANOVA was conducted if the normality was satisfied. Kruskal-Wallis H test was conducted instead if the normality was violated. Differences were determined using Tukey post hoc analysis if the difference among groups were statistically significant. In all tests, significant effects/interactions were those with a *P* value of <0.05. All statistical analyses were performed using SPSS 20.0 software.

Pearson’s correlation was used in analyzing the correlation between species number, density, biomass and environmental variables. The original data were standardized by log(x + 1) transformation to satisfy the normality^65^.

Based on the log(x + 1) transformation of dominant species density and environmental variables (except for the pH), detrended correspondence analysis (DCA) was used to analyze their distribution patterns. Direct ordination was either by canonical correspondence analysis (CCA) or redundancy discriminate analysis (RDA), depending on the length of the DCA axis (where an axis of >4 = CCA and an axis of <4 = RDA).

### Data availability

The datasets generated during and/or analysed during the current study are available from the corresponding author on reasonable request.
